# Economics of Pediatric Cancer in Four Eastern Mediterranean Countries: A Comparative Assessment

**DOI:** 10.1200/GO.20.00041

**Published:** 2020-07-22

**Authors:** Adrian Gheorghe, Kalipso Chalkidou, Omar Shamieh, Tezer Kutluk, Fouad Fouad, Iyad Sultan, Richard Sullivan

**Affiliations:** ^1^ Global Health and Development, Imperial College London, London, United Kingdom; ^2^ Center for Global Development Europe, London, United Kingdom; ^3^ Center for Palliative and Cancer Care in Conflict, Department of Palliative Care, King Hussein Cancer Center, Amman, Jordan; ^4^ Department of Pediatric Oncology, Hacettepe University, Ankara, Turkey; ^5^ Faculty of Health Sciences, Global Health Institute, American University of Beirut, Beirut, Lebanon; ^6^ Department of Pediatric Oncology, King Hussein Cancer Center, Amman, Jordan; ^7^ Institute for Cancer Policy and Conflict & Health Research Group, King’s College London, London, United Kingdom

## Abstract

**PURPOSE:**

Cancer is a leading cause of death among children in the Eastern Mediterranean region, where conflict and economic downturn place additional burden on the health sector. In this context, using economic evidence to inform policy decisions is crucial for maximizing health outcomes from available resources. We summarized the available evidence on the economics of pediatric cancer in Jordan, Lebanon, the occupied Palestinian territory, and Turkey.

**METHODS:**

A scoping review was performed of seven academic databases and gray literature pertaining to pediatric cancer in the four jurisdictions, published between January 1, 2010, and July 17, 2019. Information was extracted and organized using an analytical framework that synthesizes economic information on four dimensions: the context of the health system, the economics of health care inputs, the economics of service provision, and the economic consequences of disease.

**RESULTS:**

Most of the economic evidence available across the four jurisdictions pertains to the availability of health care inputs (ie, drugs, human resources, cancer registration data, and treatment protocols) and individual-level outcomes (either clinical or health-related quality of life). We identified little evidence on the efficiency or quality of health care inputs and of pediatric cancer services. Moreover, we identified no studies examining the cost-effectiveness of any intervention, program, or treatment protocol. Evidence on the economic consequences of pediatric cancer on families and the society at large was predominantly qualitative.

**CONCLUSION:**

The available economic evidence on pediatric cancer care in the four countries is limited to resource availability and, to an extent, patient outcomes, with a substantial gap in information on drug quality, service provision efficiency, and cost-effectiveness. Links between researchers and policymakers must be strengthened if pediatric cancer spending decisions, and, ultimately, treatment outcomes, are to improve.

## INTRODUCTION

Globally, the burden of childhood cancer falls disproportionally on low- and middle-income countries.^1^ In the Eastern Mediterranean region (EMR), with its young demographic profile, cancer is the third-leading cause of death (after injuries and violence) among children aged 5 to 14 years.^2^ Most of what is known about the economics of childhood cancer is relatively recent, informed by data collected in high-income countries, and suggests there is much more to the subject than the direct cost of treatment. Indirect costs, including travel to and from hospitals, accommodation and loss of earnings, are at least as substantial as direct costs. Having a child with cancer who requires long-term (in some cases, up to 2 years) treatment may lead to job disruption, loss of income for both parents (particularly mothers), or even divorce—all of which bring about or accentuate financial hardship, including selling assets, and affect lifestyle.^3-5^ Furthermore, children surviving cancer will grow into adulthood with substantially increased risk for a range of chronic conditions such as hearing loss and abnormal lung function as a result of therapy.^6^ At the same time, there appears to be little research on the comparative value of therapeutic strategies for childhood cancers.^7^

**Context**

**Key Objective**
To summarize the evidence on the economics of pediatric cancer in Jordan, Lebanon, the occupied Palestinian territory, and Turkey.
**Knowledge Generated**
Much more is known about the availability of inputs, such as medication, human resources, and registry data, than about how efficiently, effectively, cost-effectively, or equitably inputs are being converted into services and health outcomes. Some evidence gaps are common to all four settings, such as information on the financial protection implications of accessing cancer services, whereas others are unique to each setting.
**Relevance**
Improving the quality of pediatric cancer spending decisions in the region requires conducting detailed cost analyses of pediatric cancer interventions; investing in building or, if already available, auditing cancer registries; conducting cost-effectiveness analyses of tracer interventions; and establishing governance mechanisms that promote the production and use of (locally generated) evidence in decision-making.

The relevance of these issues—cost-effectiveness, affordability, financial protection, quality of care, and long-term follow-up of survivors—is further exacerbated in health systems facing the consequences of conflict, political instability, severely constrained resources, and weak governance. In these contexts, economic evidence is even more important when informing the design and implementation of effective policy instruments (eg, cancer control plans, financing mechanisms, procurement systems).

In this study, we summarize the available evidence on economic aspects of pediatric cancer in four EMR countries to identify evidence gaps and research priorities. We focus on Jordan, Lebanon, the occupied Palestinian territory (OPT), and Turkey because these are focal countries of the Research for Health in Conflict in Middle East and North Africa project, which aims to build capacity for health research, including health economics and the political economy of health, in the region; these four countries also are the EMR’s most significant hosting nations for children with cancer. Our expectation is that such a comprehensive review will be useful for researchers and decision makers with an interest in improving pediatric cancer care outcomes in the EMR.

## METHODS

### Analytical Framework

We distilled elements from several available frameworks exploring the economics of illness^8-11^ into a framework that synthesizes economic information on four aspects: the context of the health system, the economics of health care inputs, the economics of service provision, and the economic consequences of disease (Table 1). Each domain comprises several dimensions with associated generic indicators. For example, the economics of health care inputs aspect comprises drugs, equipment, human resources, data, and treatment protocols.

**TABLE 1:**
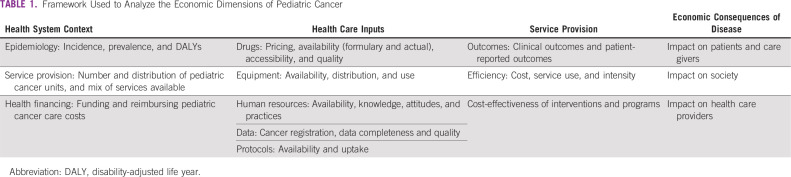
Framework Used to Analyze the Economic Dimensions of Pediatric Cancer

### Approach and Data Sources

We conducted a scoping review of the academic and gray literature pertaining to pediatric cancer in Jordan, Lebanon, OPT, and Turkey published between January 1, 2010, and July 17, 2019. We used a systematic literature search of eleven databases (Medline, Embase, Global Health, Health Management Information Consortium, PsycInfo, Scopus, Web of Science, Econlit, Index Medicus for the Eastern Mediterranean Region, and Centre for Reviews and Dissemination NHSEED and HTA databases). Full details of the search strategy are listed in Appendix Table A1. We also searched the websites of the WHO and United Nations High Commissioner for Refugees (UNHCR) for additional reports or links to relevant information sources.

#### Analysis.

Records were downloaded in Mendeley reference management software and screened by one researcher against the domains, dimensions, and generic indicators of the analytical framework. The themes and findings of relevant records were synthesized narratively and validated with academic experts in each country.

## RESULTS

### Context

#### Epidemiology.

**FIG 1:**
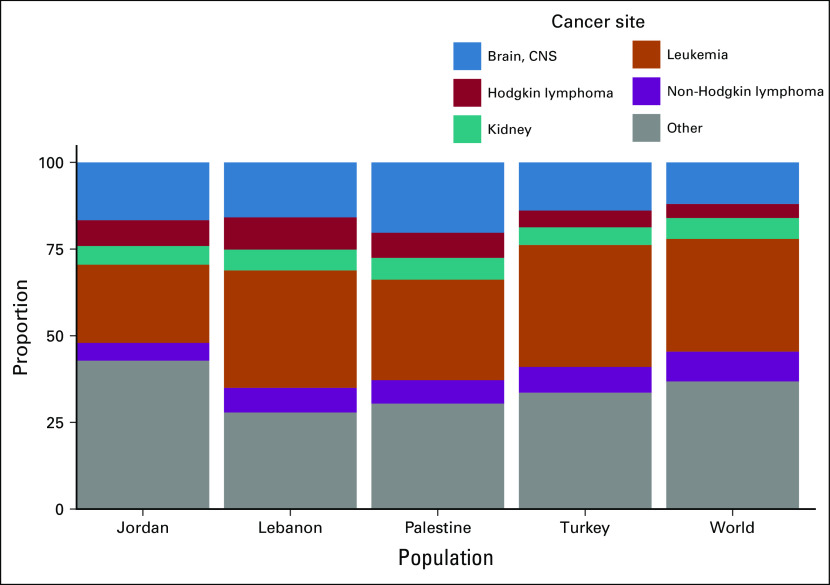
Distribution of incident cancer cases by population and cancer site (0-14 years). Source: GLOBOCAN estimates for 2019.^12^

Every year, approximately 4,000 to 4,500 new pediatric cancer cases (children aged 0 to 14 years) are currently diagnosed in Turkey, Jordan, Lebanon, and the OPT combined (Table 2). The majority of pediatric cancer cases occur in Turkey, where crude and age- and sex-adjusted incidence rates (17.2 and 17.7 per 100,000, respectively^12^) are higher than in the other three countries and > 50% higher than the world averages (10.2 and 10.3 per 100,000, respectively). The proportion of new pediatric cancer cases relative to the total number of new cancer cases (all ages) varies significantly but is higher than the 1.1% to 1.2% global average. The top five cancer site distributions are comparable across the four populations and with global figures (Fig 1), except in Jordan, where the incidence of leukemia is slightly lower than in the other countries.

**TABLE 2:**
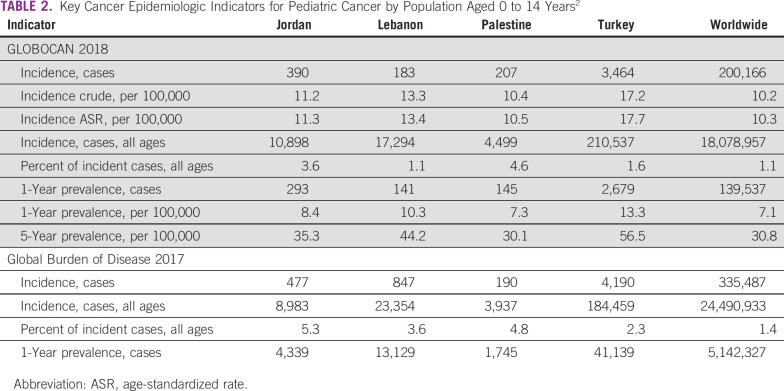
Key Cancer Epidemiologic Indicators for Pediatric Cancer by Population Aged 0 to 14 Years^2^

Some of these figures can be corroborated with registry data. For example, in Jordan, 245 new pediatric cancer cases were registered among Jordanian children in 2015, representing 4.4% of total new cancer cases.^13^ Estimates of incident cases among child refugees in the four countries are much sparser in the absence of comprehensive cancer surveillance programs and population-based registration. In Jordan, 178 cases were registered among non-Jordanian child refugees in 2015^13^ and an estimated 62 new cancer cases occur among Syrian child refugees each year.^14^ In Turkey, there are reports of 212 cases treated between 2012 and 2015 at 17 centers in 10 cities,^15^ but these probably represent only a fraction of the total. Other estimates provide institutional perspectives; for example, 623 Syrian children received treatment at Ankara Child Health and Diseases Hospital between January 2016 and August 2017,^16^ and approximately 300 Syrian children received treatment at the Children’s Cancer Center in Beirut.^17^

#### Service provision.

Pediatric cancer care is provided in departments or units of generalist, cancer, or pediatric hospitals. In Lebanon, there are 14 hospitals with pediatric oncology departments, of which 11 are in Beirut and none are in Beqaa and South Lebanon provinces, where approximately half the Syrian refugees living in the country are located.^17^ Lebanon, however, hosts the only standalone pediatric cancer hospital in the four jurisdictions: Children’s Cancer Center of Lebanon, which opened in 2002 and is hosted at the American University of Beirut in partnership with St Jude’s Children’s Hospital in Memphis, Tennessee.

In Jordan, pediatric cancer services are provided in the oncology departments of King Hussein Cancer Center (KHCC), which sees an estimated 80% of pediatric cancer cases nationally,^14^ and of the Royal Medical Services Pediatric Hospital, as well as the pediatric departments of King Abdullah University Hospital and Princess Rahma Hospital.^19^

In the OPT, cancer is the main reason for referral outside Ministry of Health facilities in both the West Bank and Gaza,^20^ with Augusta Victoria Hospital in East Jerusalem being the main referral destination for patients with cancer and the only hospital in the OPT with a radiation facility. Patients needing to leave the West Bank or Gaza Strip for treatment face the hurdle of obtaining a permit from Israeli authorities, with a 61% average permit approval rate for patients with cancer, which is slightly higher than the average 54%. Huda Al Masri pediatric cancer department at Beit Jala Hospital and the Pediatric Oncology Unit of Al-Najah University in Nablus serve patients in the south and north parts of the West Bank, respectively. A pediatric oncology unit was recently established in the Gaza Strip by the Palestine’s Children Relief Fund.

Regarding services other than cancer therapy (eg, palliative care, home care, long-term support), most sources refer to palliative care being underdeveloped.^21-23^ Only one study explored in some depth potential barriers or challenges for palliative care: a survey of heads of 31 pediatric oncology units in Turkey reported the lack of professionals (58%), lack of physical space (48%), and lack of education (26%) among the perceived challenges to the development of palliative care.^24^ We found one study reporting on the availability of services other than palliative care: a survey of 33 pediatric oncology departments in Turkey.^25^ The authors found that only four units offered long-term follow-up of cancer survivors; the ones that did not invoked the lack of a survivorship clinic, insufficient education on survivorship, and busy work schedules.^25^ KHCC appears to have the most developed comprehensive pediatric palliative care in Jordan and the region, with inpatient and outpatient consultation service, inpatient hospice, and home care.^26^

#### Financing.

The health financing systems across the four jurisdictions have similarities and differences, as reflected by key indicators (Table 3). Although the extent to which health is a domestic spending priority, reflected in the proportion of domestic government health expenditure from general government expenditure, is comparable across the four settings (range, 10%-16%), health spending per capita (range, USD 257-645), population coverage with some form of insurance (range, 42%-99%), and the reliance on out-of-pocket payments (range, 16%-46% of current health expenditure) are markedly different. In Lebanon, in particular, the combination of high spending levels, low insurance coverage, and high out-of-pocket payments raises issues of protection against catastrophic health spending.

**TABLE 3:**
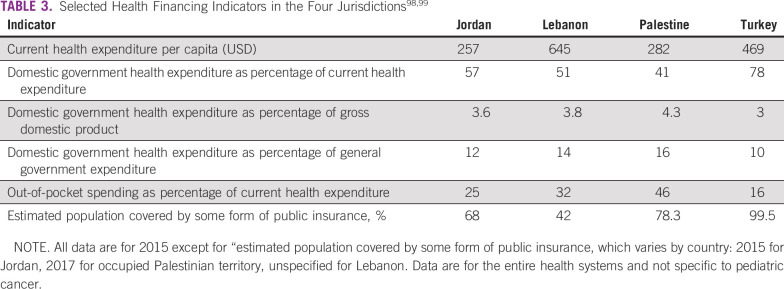
Selected Health Financing Indicators in the Four Jurisdictions^98,99^

Cancer care is free for children in Jordan^19^ and Turkey.^27^ In Lebanon, medicines for life-threatening diseases, including cancer, are provided for those without insurance coverage through an independent financing scheme overseen by the Ministry of Public Health (MOPH).^28^ Private financing initiatives, such as Acute Support for Children and Adolescents in Lebanon, Amel Association International, and American Lebanese Syrian Associated Charities/St Jude Children’s Research Hospital play an important role in fundraising to support access for refugee families who cannot afford treatment.

The situation of children with cancer who are Syrian refugees (as well as other migrant populations) is radically different across the four jurisdictions. In Turkey, the costs of cancer care for Syrian child refugees is covered by the government.^29^ In Jordan, the government initially allowed Syrians registered with UNHCR to access health care services free of charge in Ministry of Health’s facilities, but as of November 2014, Syrian refugees became required to pay the noninsured Jordanian rate for all types of health services in public facilities.^14^ On the other hand, the UNHCR selectively funds expensive treatments according to the decision of the UNHCR Exceptional Care Committee. A previous study of the committee’s decisions in Syria and Jordan between 2009 and 2012 showed that 48% of applications were funded, and the major reason for application denial was a determination of a poor prognosis.^30^ More than two-thirds of Syrian children are covered through charity funds organized by the King Hussein Cancer Foundation for treatment at KHCC, where Syrian children receive the same treatment as Jordanian patients.

### Economics of Health Care Inputs

#### Drugs.

We could not identify any analysis that referred specifically to the availability, pricing, reimbursement, procurement, quality, or dispensing of drugs used in pediatric cancer in these four countries. However, we were able to construct a picture of the availability and accessibility for 10 of 19 anticancer drugs on the 2015 WHO Model List of Essential Medicines for Children,^31^ based on data collected throughout 2015 in a survey of 82 non-European countries, including Jordan, Lebanon, Palestine, and Turkey^32^ (Table 4). Although all 10 drugs are on the national drug lists of each of the four countries with full reimbursement (with the exception of cyclophosphamide tablets in OPT), some of them are not always available, particularly in Lebanon and Palestine, and the information is missing for Jordan. Barriers to accessibility differ by country: they tend to be manufacturing problems or lack of commercial motive in Turkey, budget restrictions in Palestine (for most cancer drugs), and no reliable supplier in Jordan. For Gaza, in particular, there are consistent reports of severe shortages of cancer drugs^33-35^ but no published systematic inventory.

**TABLE 4:**
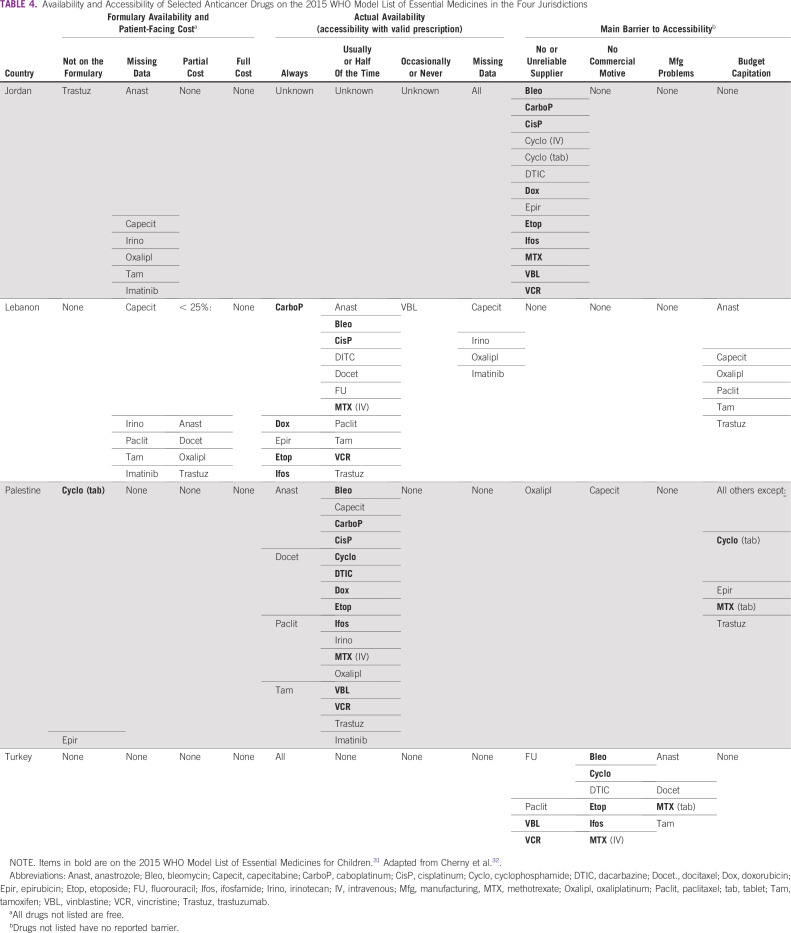
Availability and Accessibility of Selected Anticancer Drugs on the 2015 WHO Model List of Essential Medicines in the Four Jurisdictions

We identified only one study directly comparing publicly listed prices for 22 anticancer drugs; the authors found prices in Lebanon tended to be among the lowest compared with the other eight countries (seven Asian countries and Egypt) in that study.^36^ There are indications of cancer drug prices decreasing since 2011 in Lebanon, including through procurement negotiations led by the MOPH.^37^ More recently, the government of Jordan reduced in June 2019 the list prices of > 1,000 drugs, including a 10% reduction for cancer drugs.^38^ We did not identify any study examining the quality of drugs for pediatric cancer in the four countries reviewed in our study.

#### Equipment.

We could not identify any systematic assessment regarding the availability, procurement processes, or use rates of therapeutic and diagnostic equipment in pediatric oncology units across the four jurisdictions. A global analysis underpinned by 2012 data found the available radiotherapy equipment in Jordan, Lebanon, and Turkey met the estimated demand for cancer care (at least at the time)^39^; a previous analysis for the Lancet Oncology Commission also did not identify significant shortages in radiotherapy capacity.^40^ In the Palestinian health sector, in 2014, three of 35 hospitals had a magnetic resonance imaging scanner—all three were private hospitals.^41^

#### Human resources.

A single study provided in-depth staffing information in pediatric oncology units. This was a 2012 survey of 31 pediatric oncology departments in Turkey that found all units had one or more pediatric oncology doctors and nurses, but only 26%, 13%, 7%, and 7% had a psychologist, social worker, psychiatrist, and physiotherapist, respectively.^24^ Other sources provided only descriptive information or included only the total number of pediatric oncologists; for example, as of 2012, there were 15 pediatric oncologists working in Lebanon,^23^ 20 in Jordan,^19^ and approximately 70 in Turkey.^27^

#### Treatment protocols.

We could not identify any source assessing which treatment protocols are used in pediatric oncology departments across each of the four jurisdictions. However, a number of clinical outcomes studies refer to the use of a range of international protocols, with or without adaptations, such as Berlin-Frankfurt-Munster,^42,43^ Children’s Cancer Group, Children’s Oncology Group,^44,45^ St Jude Total,^46,47^ National Wilms Tumor Study^48^; or national protocols (eg, in Turkey).^49,50^ Turkey appears to be the only country in the sample where a national-level professional association (Turkish Pediatric Oncology Group) produces treatment protocols and guidelines, and conducts studies to assess their outcomes.

#### Cancer registration.

A number of sources describe in some detail pediatric cancer registration in Turkey,^51,52^ Jordan,^53^ and Palestine.^54^ However, only the Palestinian source refers explicitly to the performance of cancer registration, specifically its fluctuations across space (eg, under-registration in selected districts) and time (eg, deficient leukemia registration between 2003 and 2005).

We found no studies attempting to estimate the extent of childhood cancer underdiagnosis or underreporting. However, a Turkish study examined the determinants of time to diagnosis (from contact with a health professional) and found longer time to diagnosis when the first contact was a nonpediatric specialist and much shorter as well as for infants and children older than 10 years.^55^

### Economics of Service Provision

#### Efficiency.

We found no studies examining the efficiency or productivity of pediatric cancer services or facilities, but we identified three studies estimating the cost of specific pediatric cancer interventions. One Turkish study analyzed data from the national social security system for 148 pediatric cancer cases across five cancer types (Hodgkin lymphoma, non-Hodgkin lymphoma, leukemia, medulloblastoma, and osteosarcoma) and estimated an average cost of USD 25,045 per patient.^56^ Two studies in Jordan estimated the average cost of chemotherapy for children with Ewing sarcoma^57^ and of two l-asparaginase formulations for acute lymphoblastic leukemia (ALL),^58^ respectively.

#### Cost-effectiveness.

We could not identify any full economic evaluation of a pediatric cancer health intervention or program.

#### Patient outcomes.

We identified two nationwide analyses of survival after cancer treatment, both in Turkey. An analysis of data from 2002 to 2008 in the Turkish National Pediatric Cancer Registry found an overall 5-year survival rate of 65%,^52^ and a later analysis of the same registry (2009 to 2018 data) found an overall 5-year survival rate of 70.8%.^51^ Other studies were either conducted at a single hospital and/or focused on a single type of cancer. For example, in Turkey, studies focused on ALL,^45,59^ neuroblastoma,^50^ rhabdomyosarcoma,^60^ or Ewing sarcoma.^61^ In studies of data from Jordan, a 3-year overall survival rate of 75% was reported for all pediatric cancer types at King Hussein Cancer Center^62^ including ALL^63^ and CNS tumors^64^. Study data from Lebanon focused on osteosarcoma^44^ and Ewing sarcoma.^65^ Most studies concluded that the reported survival rates were generally lower but nevertheless comparable to those observed in high-income countries.

We could find only one study that applied a generic health-related quality-of-life instrument, namely the Short Form Survey–36, to evaluate the quality of life of children with cancer and their parents in Turkey. According to the study findings, they had affected health status relative to control subjects.^66^ Other studies documented the quality of life of children undergoing cancer treatment,^67-70^ of parents and caregivers,^18,71^ and of child survivors.^72,73^ Many more studies examined the psychometric properties of various disease-specific instruments, particularly in Turkey, such as the KINDL scale,^74^ Quality of Life and FAMCARE scales among caregivers,^75^ Children's International Mucositis Evaluation Scale,^76^ or the Adolescent Pediatric Pain Tool^77^; and Jordan, including the Pediatric Quality of Life Inventory (PedsQL) Generic Core Scale, PedsQL Family Impact Scale, and the PedsQL Healthcare Satisfaction Hematology/Oncology Scale.^78^

### Economic Consequences of Disease

#### Economic burden on health service providers and the health care system.

We identified one study protocol referring specifically to the costs of pediatric cancer care, and that was in Jordan.^79^ Other sources referred to the overall financial burden of cancer care, for example, from the perspective of the Lebanese MOPH (cancer drugs cost USD 140 million over 3 years^36^); but the way the data were presented did not allow disaggregation.

#### Economic burden on individuals, care givers, and families.

Although a number of sources mentioned the financial burden of cancer care for parents and families (eg, in relation to the cost of chemotherapy drugs in Lebanon^23^) or the relationship between financial concerns and measures of well-being,^80-82^ we identified a single study that attempted to quantify this burden (n = 85 parents or care givers) on a scale from 1 (low) to 10 (high) and reported an average score of 5.35 (standard deviation, 2.60), suggesting moderate financial hardship.^83^ One qualitative study in Jordan, informed by interviews with 12 mothers and 12 fathers of children with cancer, documented what some Jordanian families may experience in terms of financial burden (eg, losing work, transportation, hotel costs).^18^ We found no study that quantified the volume of out-of-pocket payments, prevalence and distribution of coping mechanisms, or impact on job loss and job productivity.

### Summary of Findings

Our main findings are summarized narratively in Table 5. Across the four countries, most of the available economic evidence pertains to the availability of health care inputs (ie, drugs, human resources, cancer registration data, and treatment protocols) and, to an extent, individual-level outcomes, either clinical or health-related quality of life. We identified little evidence on the efficiency or quality of inputs of pediatric cancer services; also, we identified no studies that examined the cost-effectiveness of an intervention, program, or treatment protocol. There was also limited information, most of it qualitative, on the economic consequences of pediatric cancer on families and the society at large.

**TABLE 5:**
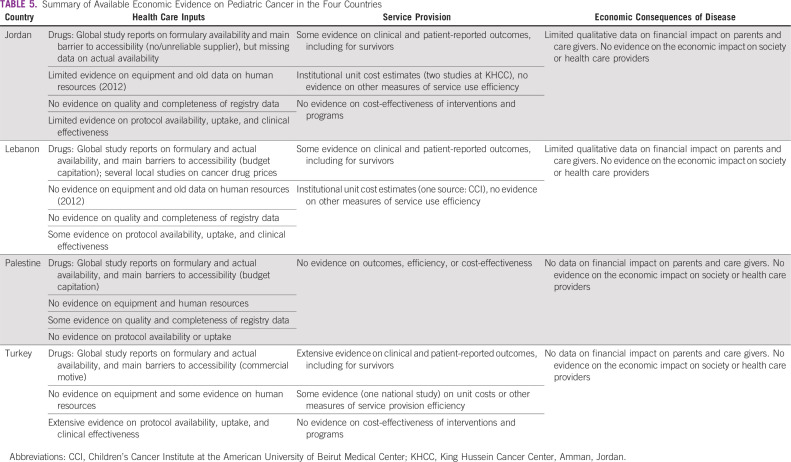
Summary of Available Economic Evidence on Pediatric Cancer in the Four Countries

### Interpretation of Findings

Our findings point toward substantial gaps in the available economic evidence on pediatric cancer in all four countries. Some of these gaps are common to all, such as information on cost-effectiveness or implications for financial protection, whereas others are unique to each setting—for example, the availability and prices of cancer drugs in Jordan.

Explaining the evidence gaps is not straightforward, because several factors may be at play simultaneously. One may be a reluctance to engage with pediatric cancer from an economic perspective, given the relatively small number of cases (up to 5% of new cancer cases), the moral imperative of realizing children’s right to health, and the risk of any economic argument being construed as rationing or cost cutting. Indeed, it can be counterargued that leaving economics out of the picture can easily lead to suboptimal decisions that deprive unseen patients of care they could have received. This counterargument is at the heart of framing childhood cancer care within the universal health care agenda.

The misalignment between research and policy priorities, reflected in the absence of coherent health research strategies in the region,^84^ may also explain the gaps. The immediate example is drug pricing. Despite limited evidence on drug prices and their impact on the affordability of cancer care (to third-party payers and families) or efficiency of service delivery in general, the governments of Lebanon and Jordan have directed their attention to the issue over the past years and reduced list prices, presumably in response to perceiving drug prices as major cost drivers.

There is also the issue of limited capacity for health economics in the four countries, EMR and the Middle East and North Africa more broadly, which may have prevented health economics analyses from being conducted. Previous research programs, such as Research Capacity for Public Health in the Mediterranean, have tried to address this,^85^ but much more is to be done.

### Implications for Research and Policy

For researchers and policymakers, our findings point to the importance of local economic evidence to generate context-specific insights, particularly in the context of action for progress toward universal health coverage: Although some evidence is available and can be used already (eg, on specific barriers to cancer drugs accessibility), a substantial amount of information on current pediatric cancer provision is lacking, particularly on the main drivers of the cost of care, quantitative measures of family-level financial impacts, and cost-effectiveness of treatment protocols. This precludes making informed decisions on how to expand coverage and quality of care in the context of the WHO Global Initiative on Childhood Cancer^86^ and the 2017 World Health Assembly resolution on cancer.^87^

We propose four areas for additional consideration with associated recommendations:

1. Costs: Conduct detailed cost analyses of pediatric cancer services from the perspectives of providers and users as starting points for improving the quality, efficiency, and equity of cancer care provision. There is already evidence that quality cancer care can be delivered in low-resource settings at a fraction of the cost in high-income settings.^88^
2. Health outcomes: Invest in building cancer registries and, where these are available, auditing and validating their data for pediatric cancers. There is evidence that accuracy of general cancer registries differs between adult and pediatric cancers because the latter usually constitute a more heterogeneous group.^89,90^
3. Cost-effectiveness: Conduct pilot cost-effectiveness analyses of tracer interventions as the starting point for a multisectoral conversation on demonstrating and improving the value of cancer care. Initial analyses can focus on small, well-defined units (eg, Tanzania,^91^ Mexico,^92^ El Salvador^93^).
4. Governance: Establish a mechanism that promotes the production and use of evidence for cancer decisions by bringing knowledge producers, knowledge brokers, and knowledge users together. Such an example could be India’s National Cancer Grid,^94^ particularly for improving the quality of pathways and models of care. The costs (direct and indirect) of treating children with cancer need to be equitable, addressed by UNHCR and direct healthcare assistance to support host countries, particularly because pediatric oncology in refugee populations is an additional burden on health care systems beyond their own domestic populations. One aspect worth looking at is ensuring that reimbursement (at least from public funds) is tied to validated assays certifying the quality of anticancer drugs.

### Strengths and Limitations

The main limitations of our analysis relate to the absence of a systematic assessment based on inclusion and exclusion criteria and the absence of a quality-assessment component; these were not attempted for practical reasons in light of our policy-facing objective and of the considerable difficulties raised by the wide range of economic topics surveyed, each with its methodological quality checklists. To our knowledge, this is the first analysis focused on the economics of pediatric cancer in the EMR and, for that matter, globally. It complements two previous analyses that either had a regional focus on cancer epidemiology in the general population,^95,96^ or a global focus on childhood cancer,^1^ but neither included a discussion of economic implications.

In conclusion, the available economic evidence on pediatric cancer care in the four countries is largely limited to resource availability and, to an extent, patient outcomes, with a substantial gap in evidence for drug quality, service-provision efficiency, and cost-effectiveness. Links between researchers and policymakers must be strengthened to identify context-specific priorities for generating and using appropriate economic evidence that can improve pediatric cancer–spending decisions and, ultimately, treatment outcomes.

## Figures and Tables

**TABLE A1:**
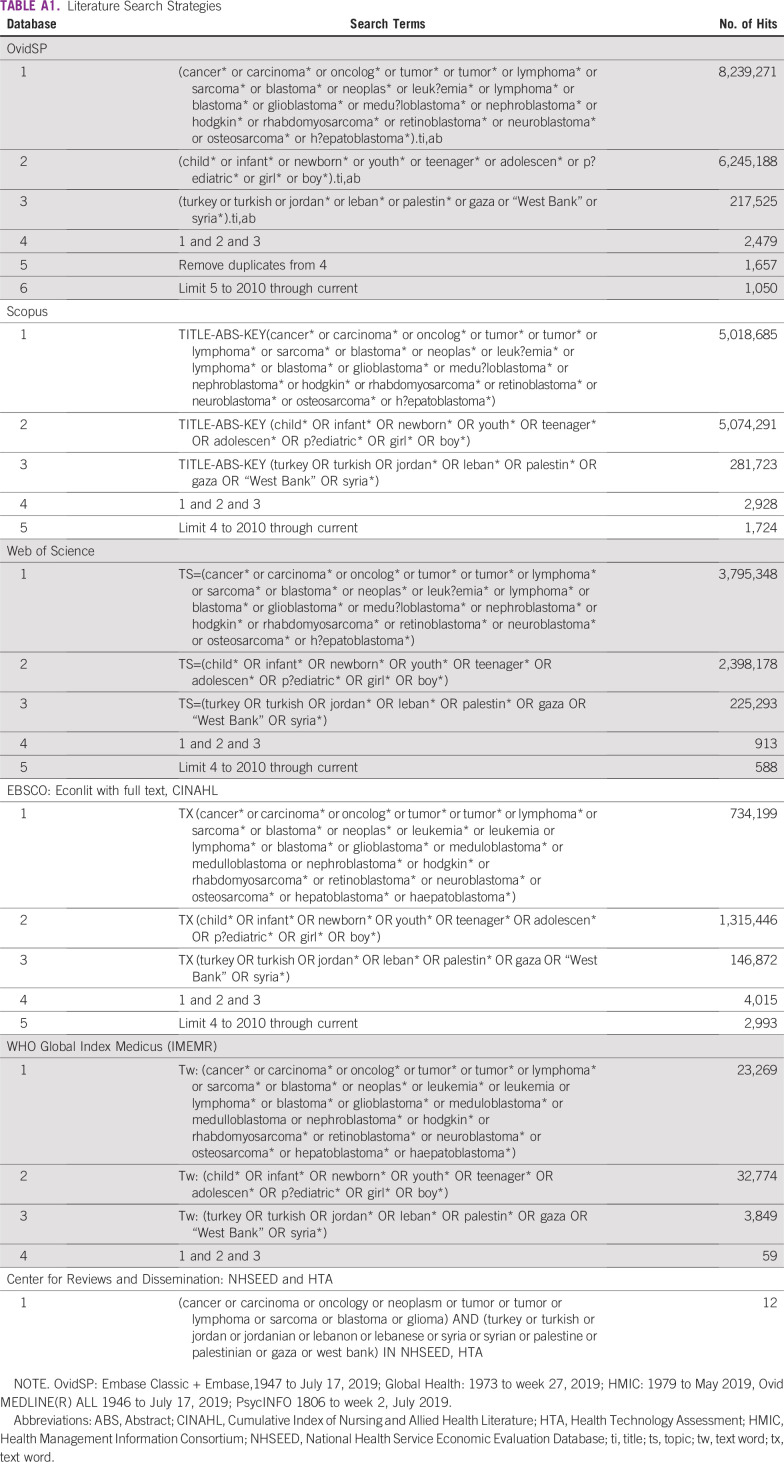
Literature Search Strategies
